# Impact of Radiofrequency Exposure From Mobile Phones on the Risk of Developing Brain Tumors in Korean and Japanese Adolescents: A MOBI-Kids Case-control Study

**DOI:** 10.2188/jea.JE20230005

**Published:** 2024-04-05

**Authors:** Noriko Kojimahara, Yong-Han Lee, Ae-Kyoung Lee, Sanghyuk Bae, Ho-Jang Kwon, Mina Ha, Yasuto Sato, Masao Taki, Joe Wiart, C.E. Langer, Elisabeth Cardis

**Affiliations:** 1 Section of Epidemiology, Shizuoka Graduate University of Public Health, Shizuoka, Japan; 2 Department of Preventive Medicine, College of Medicine, Dankook University, Chungnam, Republic of Korea; 3 Radio Technology Research Department, Electronics and Telecommunications Research Institute (ETRI), Daejeon, Republic of Korea; 4 Department of Preventive Medicine, College of Medicine, The Catholic University of Korea, Seoul, Republic of Korea; 5 Electromagnetic Compatibility Laboratory, National Institute of Information and Communications Technology, Tokyo, Japan; 6 Faculty of System Design, Tokyo Metropolitan University, Tokyo, Japan; 7 Laboratoire de Traitement et Communication de l’Information (LTCI), Telecom Paris, Institut Polytechnique de Paris, Palaiseau, France; 8 Barcelona Institute for Global Health (ISGlobal), Barcelona, Spain; 9 Pompeu Fabra University, Barcelona, Catalonia, Spain; 10 Spanish Consortium for Research and Public Health (CIBERESP), Instituto de Salud Carlos III, Madrid, Spain

**Keywords:** radiofrequency exposure, mobile phones, brain tumors, case-control study, adolescents

## Abstract

**Background:**

This study aimed to examine the association between risk of brain tumors and radiofrequency (RF) exposure from mobile phones among young people in Korea and Japan.

**Methods:**

This case-control study of brain tumors in young people was conducted in Korea and Japan under the framework of the international MOBI-Kids study. We included 118 patients diagnosed with brain tumors between 2011 and 2015 and 236 matched appendicitis controls aged 10–24 years. Information on mobile phone use was collected through face-to-face interviews. A detailed RF exposure algorithm, based on the MOBI-Kids algorithm and modified to account for the specificities of Japanese and Korean phones and networks, was used to calculate the odds ratios (ORs) for total cumulative specific energy using conditional logistic regression.

**Results:**

The adjusted ORs in the highest tertile of cumulative call time at 1 year before the reference date were 1.61 (95% confidence interval [CI], 0.72–3.60) for all brain tumors and 0.70 (95% CI, 0.16–3.03) for gliomas, with no indication of a trend with exposure. The ORs for glioma specifically, were below 1 in the lowest exposure category.

**Conclusion:**

This study provided no evidence of a causal association between mobile phone use and risk of brain tumors as a whole or of glioma specifically. Further research will be required to evaluate the impact of newer technologies of communication in the future.

## INTRODUCTION

In recent decades, radiofrequency (RF) exposure has increased among the general population with the increased use of mobile phones and communication technologies. RF electromagnetic fields (EMFs) from mobile phones have been classified by the International Agency for Research on Cancer^1^ as group 2B (possibly carcinogenic to humans). Despite public concerns regarding the possible health impacts of EMFs generated by mobile phones, epidemiologic studies have not yet conclusively demonstrated these effects.^2^^–^^5^ Since the source of RF-EMF exposure is near the head during a mobile phone call, the World Health Organization (WHO) recommended investigating whether the use of mobile phones affects the health of children.^6^ Brain tumors are the second leading cause of all mortality^7^ after leukemia in people younger than 25 years.

Children aged 5–8 years reportedly have higher specific absorption rates (SARs) in the brain compared with adults^8^^,^^9^ because of their anatomical characteristics. Although adolescence have similar SARs to adults, exposure in childhood may lead to a higher risk of brain tumors later in life, especially because young people start using mobile phones at a younger age compared with adults.^10^

The results of the international MOBI-Kids study were recently published,^5^ providing no evidence of a causal association between wireless phone use and brain tumors in young people. Mobile phone models and the mobile communication technologies differed in Korean and Japan from those in most other countries during the study period of MOBI-Kids study. This study, therefore, presents results of specific analyses of brain tumor risk in the Korean and Japanese subsets of MOBI-Kids (referred throughout as the KJ study), taking into account the characteristics of the networks and phones in these countries. We also included brain tumors irrespective of their anatomical location, unlike the main MOBI-Kids analyses, which excluded tumors in the mid-brain.

## METHODS

### Participants and methods compared with those in the MOBI-Kids international study

The detailed MOBI-Kids methods have been published elsewhere.^11^ Briefly, in Korea and Japan, all inpatient cases of brain tumors among patients aged 10–24 years diagnosed between 2011 and 2015 at 15 participating Korean hospitals in Seoul, Incheon, and Chungcheongnam and 31 participating Japanese hospitals in the Tokyo Metropolitan area were eligible for inclusion in this study, regardless of the anatomical location of their tumors, unlike in the full MOBI-Kids study. The 5th edition of the WHO Classification of Tumors of the Central Nervous System published in 2021 incorporated the role of molecular diagnostics^12^; however, we adopted the 3rd edition^13^ due to the timing of the study period.

Two controls were post-hoc matched to age (within 1.5 years for patients aged less than 17 years at diagnosis and within 2.5 years for older patients), area of residence, and sex with patients diagnosed with acute appendicitis who had undergone laparotomy or surgical appendectomy in general surgical departments of the participating hospitals. To be eligible, control participants must have undergone surgery within 6 months of the first imaging diagnosis and be interviewed between, before, and after 1 year of the interview date of the case to which they were matched, in line with the international study. Participants and potential controls were approached by the clinical coordinators of each participating hospital, who provided information about the study.

The interviews with the potential participants and controls were arranged after verifying the patients’ or their parents’/guardians’ consent to participate. Personal, computer-assisted interviews were conducted by experienced, trained interviewers. We investigated mobile phone use, including information regarding the start and end times of use for each device, average time per call, average number of calls, and changes during the use period. All exposure variables were calculated up to 1 year before the reference date, which was defined as the date of diagnosis for patients and date of appendectomy for controls. Using XGridmaster^14^ software, the tumor location was identified in 1-cm cubes on a three-dimensional (3D) grid of the brain. Regular mobile phone use was defined as use at least once a week for a period of 3 months or more in accordance with the international MOBI-Kids protocol.^15^

The inclusion criteria differed in the international MOBI-Kids study in that tumors originating in the middle of the brain, where little RF energy deposition from wireless phones is expected, were excluded, although such tumors were included in this study. We also included additional controls from outside the Tokyo metropolitan area who were excluded in the international study, in which participants were matched on restricted geographical areas of residence. The exclusion criteria were same as the international MOBI-Kids study: insufficient knowledge of the language(s) and/or neurofibromatosis.

This study was approved by the Institutional Review Boards of Dankook University (approval number: 0423, 11/2011) and Tokyo Women’s Medical University (approval number: 2394, 8/2011). All participants, parents, or guardians provided written informed consent for participation in the study.

### Algorithm for estimating RF-EMF exposure to the brain

The amount of RF-EMF energy absorbed in the brain from mobile phone use depends on tumor location, phone model, laterality, call frequency/duration, hands-free use, and communication system,^16^ as reported in the INTERPHONE Study.^17^ Personal Digital Cellular (PDC) as the second generation (2G) and personal handy-phone system (PHS)^18^ were used in Japan mainly in the 1990’s, while the third generation (3G) was adopted in Korea in the mid-2000s. The RF-EMF exposure levels from such networks was considerably higher than that from 3G and Long-Term Evolution (LTE).^19^

In this study, Korean and Japanese data were integrated using a unique algorithm. We calculated RF energy by location using the reference brain for each phone type and frequency band using an algorithm based on the MOBI-Kids algorithm^20^ that considered the cumulative call time, phone used, prevailing mobile phone technology at the time, and characteristics of the communication system and phone, including the adaptive power control (APC). The APC, defined as the ratio of the average transmission power to maximum power of the phone, depends on the frequency, communication system, and operator, as shown in Table 1. According to the environmental electromagnetic field measurement, RF exposure measurement were practically similar in urban and rural area in the two countries (data not shown). Hence, power ratios were estimated only for the urban area in the KJ study. In addition, the phone models reportedly used by the study participants were classified differently than in the international study^21^^,^^22^ and included phone classification specific to the types of phones used in Korea and Japan: four bar, four slide, and six flip phones (eFigure 1). As in the MOBI-Kids study, the algorithm^20^ estimated the RF-EMF dose as the total cumulative specific energy (CSE; J/kg) absorbed at a given location in the brain (eFigure 2), as a function of the phone model, communication system, and reported phone usage (eg, cumulative call time). As more than 95% of the participants reported using mobile phones in urban areas, only APC modifiers in these areas were applied in the KJ study. Because the use of headsets in our population was rare during the study period, we assumed that no modification due to headsets use was required. In addition to the history of mobile phone use, the questionnaire collected detailed information on the history of using cordless Digital Enhanced Cordless Telecommunications (DECT) phones and Wi-Fi, occupational history, or daily use, including specific questions on exposure to EMF sources.^23^ However, the KJ study algorithm calculated RF exposure exclusively from mobile phone use, including 2G, 3G, LTE, and PHS communication systems.

**Table 1.** Discontinuous Transmission (DTx) and Adaptive power control (APC) modifiers for mobile phones in the KJ study

Network	Generation or communication system	DTx	APC–Tx power ratio (avg./max.)			

			Korea		Japan	

			Urban	Service period	Urban	Service period
**CDMA2000**	2.5G	1	0.003–0.005	2002∼2021	0.005	2000∼2012

	3G	1	0.0004–0.002	2007∼	0.005	2004∼

**WCDMA**	3G	1	0.004–0.02	2007∼		

**LTE**	4G	1	—	2011∼	0.01	2014∼

**PDC**	2G	0.6	—	—	0.5	1993∼2012

**PHS**	DECT	1	—	—	1	1995∼

CDMA, Code Division Multiple Access; DECT, Digital Enhanced Cordless Telecommunications; LTE, Long Term Evolution; PDC, Personal Digital Cellular; PHS, personal handy-phone system; WCDMA, Wideband Code Division Multiple Access.

The APC for Voice over LTE was not considered in this study because LTE service of Korea launched in 2011 mostly had serviced data communication, such as text message use and internet access, in the early years.

The CSE was estimated in different locations (based on the 3D coordinates of the tumors in XGridmaster), including in the entire brain hemisphere in which the tumor was located, entire tumor, and at the tumor’s center of gravity (COG) in the patients and their matched controls.

### Statistical analysis

Descriptive analyses were conducted of the main demographic and exposure characteristics of the study subjects, by case/control status and by country. The odds ratio (OR) and 95% confidence interval (CI) were calculated using conditional multiple logistic regression analysis, with covariates of country, maternal educational level, for the risk of developing a brain tumor as a function of cumulative mobile phone use time and cumulative estimated RF energy (J/kg). The exposure variables (cumulative call time and RF CSE at the different locations in the brain) were categorized in tertiles on the basis of the distribution of these variables among the controls at 1 year before the reference date. Analyses are shown of the data from Korea and Japan together as numbers of cases were too low for meaningful comparisons of risk between countries.

All statistical analyses were performed using R version 3.5.1 (R Foundation for Statistical Computing, Vienna, Austria), with a statistical significance level of 0.05.

## RESULTS

### General characteristics of participants

Table 2 shows the baseline characteristics of the participants, including 52 from Korea, 66 from Japan, and 104 and 132 controls, respectively. In all Korean and Japanese groups, participants from urban areas outnumbered ones from rural areas but controls were more from urban area than cases. Goedhart et al reported a very low percentage of hands-free device use among reported hands-free users (comparison: self-reported vs recorded)^24^; hence we, did not consider the use of headsets as information about the rates of use for each mobile phone was missing.

**Table 2.** Characteristics of Korean and Japanese participants at 1 year before the reference date

	Cases			Controls		

	Korea (*n* = 52)	Japan (*n* = 66)	*P* values	Korea (*n* = 104)	Japan (*n* = 132)	*P* values
Gender, Men, *n* (%)	31 (59.6)	39 (59.1)	1.000	62 (59.6)	78 (59.1)	1.000
Age at diagnosis, mean (SD)	15.56 (3.47)	16.66 (4.52)	0.152	15.82 (3.69)	16.64 (4.49)	0.134
Mother’s education, *n* (%)						
High school or less	27 (51.9)	28 (42.4)	0.001	63 (60.6)	36 (27.3)	<0.001
College or vocational schools	3 (5.8)	23 (34.8)		6 (5.8)	60 (45.5)	
University or higher	19 (36.5)	11 (16.7)		33 (31.7)	32 (24.2)	
Unknown	3 (5.8)	4 (6.1)		2 (1.9)	4 (3.0)	
Urban residency^*^, *n* (%)	46 (88.5)	34 (51.5)	<0.001	78 (75.0)	115 (87.1)	<0.001

Mobile phone use, *n* (%)	50 (96.2)	39 (59.1)	<0.001	87 (83.7)	86 (65.2)	<0.001
right ear, *n* (%)	32 (61.5)	31 (47.0)	0.046	73 (70.2)	66 (50.0)	0.122
2^nd^ generation (2G)	—	4 (10.3)		—	14 (16.3)	
3^rd^ generation (3G)	32 (100)	39 (100)		87 (100)	85 (98.8)	
4^th^ generation (4G)	—	3 (7.7)		—	13 (15.2)	
PHS	—	5 (12.8)		—	16 (18.6)	

Tumor types, *n* (%)						
Glioma	21 (40.4)	25 (37.9)	0.704	—	—	—
Meningioma	3 (5.8)	2 (3.0)		—	—	
Others	28 (53.8)	39 (59.1)				

SD, standard deviation.

^*^P < 0.05 between total cases and controls, in which controls more from urban than cases.

Of the 118 brain tumors, 46 were classified as gliomas, including 3 astrocytomas, 2 ependymomas, and 1 glioblastoma. Only five cases of meningioma were included in the study. Although the category “Others” included a variety of morphologies, germinoma was the most frequent (*n* = 22), followed by ganglioglioma (*n* = 6). Given the small number of such tumors, no separate analysis was conducted for these tumor types.

### Mobile phone use at 1 year before the reference date and RF-EMF exposure estimated by the KJ study algorithm

Regular mobile phone use was substantially higher among Korean cases and controls (96.2% and 95.2%, respectively) compared with their Japanese counterparts (66.7% and 69.7%, respectively) (Table 3). To avoid consideration of RF exposure received after the development of a brain tumor, the exposure variables were calculated up to 1 year before the date of the first image showing a space-occupying lesion for patients and date of appendectomy for controls. The mean duration of mobile phone use ranged from 3.10 years in the Japanese case group to 3.95 years in the Korean case group. The number of calls was approximately 10 times higher in the Korean case group and 3.5 times in the Korean control group, compared with that in the corresponding Japanese groups. Cumulative call times showed no significant differences at 419.3, 207.9, 425.5, and 339.0 hours, respectively.

**Table 3.** Comparison between Korean and Japanese participants in terms of mobile phone use at one year before the reference date and RF energy estimated by the KJ algorithm

	Cases			Controls		

	Korea	Japan	*P* values	Korea	Japan	*P* values
Mobile phone use, yes, *n* (%)	50 (96.2)	39 (59.1)	<0.001	62 (59.6)	78 (59.1)	<0.001
Duration of mobile phone use, years, mean (SD)	3.95 (2.27)	3.10 (3.43)	0.128	3.13 (2.51)	3.26 (3.57)	0.760
Numbers of calls, mean (SD)	10,190 (12,033)	1,076 (2,210)	<0.001	9,495 (14,753)	2,173 (5,967)	<0.001
Cumulative call times, hours, mean (SD)	419.3 (649.4)	207.9 (687.6)	0.092	425.5 (938.8)	339.0 (1,690.3)	0.640
RF energy of whole hemisphere tumor side, mean (SD)	19.5 (35.6)	58.4 (173.6)	0.115	20.6 (81.7)	441.1 (2,334.9)	0.068
tumor	12.0 (28.8)	17.0 (63.8)	0.599	15.7 (104.1)	82.1 (373.2)	0.080
center of gravity	11.5 (28.2)	14.8 (61.2)	0.718	15.4 (104.1)	74.6 (345.2)	0.093

SD, standard deviation.

As estimated by the KJ study algorithm, the mean and standard deviation (SD) for the RF energy of the whole hemisphere on the tumor side was 19.5 (SD, 35.6) J/kg in the Korean case group and 20.6 (SD, 81.7) J/kg in the Korean control group. The estimated RF CSE of 441.1 (SD, 2,334.9) J/kg in the Japanese control group was higher than that in the other groups (*P* = 0.068) due to the inclusion of 14 PDC and 16 PHS users in this group and 4 and 5 users in the case group, respectively. The estimated amount of RF energy in the entire tumor was 12.0 (SD, 28.8) J/kg and 17.0 (SD, 63.8) J/kg and 15.7 (SD, 104.1) J/kg and 82.1 (SD, 372.2) J/kg in the Korean and Japanese case and control groups, respectively. The cumulative RF energy in the COG of the tumor was 11.5 (SD, 28.2) J/kg and 14.8 (SD, 61.2) J/kg in the Korean case and control groups, respectively, and 15.4 (SD, 104.1) J/kg and 74.6 (SD, 345.2) J/kg in the Japanese case and control groups, respectively.

### Mobile phone use and brain tumor risk

Table 4 shows results on all brain tumor of conditional logistic regression by tertiles of cumulative call time and RF energy. The OR in the highest tertile of cumulative call time (258.1 to 18,760 hours) for all brain tumors was 1.53 (95% CI, 0.69–3.40) compared with that of nonregular users. After adjustment for country and maternal education level,^25^ the OR in the highest tertile was 1.61 (95% CI, 0.72–3.60). The OR by level of RF energy in the entire hemisphere of the tumor side, tumor, and at COG of the tumor were also slightly higher than 1.

**Table 4.** Conditional logistic regression model analysis of the risk of all brain tumors in all participants

	Cases	Controls	Crude model			Adjusted model^*^		

	*n* = 118	*n* = 236	OR	95% CI	*P* for trend	OR	95% CI	*P* for trend
**Cumulative call time at one year before the reference date (hours)**								
Non-users	29 (24.6)	63 (26.7)		Ref.			Ref.	
0.1–54.8	27 (22.9)	58 (22.9)	1.06	(0.56–1.98)	0.732	1.10	(0.58–2.08)	0.389
56.1–254.5	29 (24.6)	59 (25.0)	1.23	(0.58–2.59)		1.32	(0.62–2.81)	
258.1–18,760	33 (28.0)	56 (23.7)	1.53	(0.69–3.40)		1.61	(0.72–3.60)	
**Hemisphere radiofrequency (RF) energy**								
Non-users	29 (24.6)	63 (26.7)		Ref.			Ref.	
Tertile 1	33 (28.0)	52 (22.0)	1.34	(0.72–2.52)	0.512	1.38	(0.73–2.60)	0.333
Tertile 2	25 (21.2)	63 (26.7)	0.84	(0.40–1.75)		0.91	(0.43–1.92)	
Tertile 3	31 (26.3)	58 (24.6)	1.13	(0.50–2.52)		1.16	(0.51–2.60)	
**Tumor RF energy**								
Non-users	29 (24.6)	63 (26.7)		Ref.			Ref.	
Tertile 1	29 (24.6)	56 (23.7)	1.14	(0.59–2.21)	0.787	1.14	(0.509–2.23)	0.523
Tertile 2	29 (24.6)	59 (25.0)	1.11	(0.54–2.28)		1.25	(0.60–2.61)	
Tertile 3	31 (26.3)	58 (24.6)	1.24	(0.58–2.68)		1.33	(0.61–2.89)	
**COG RF energy**								
Non-users	29 (8.2)	63 (26.7)		Ref.			Ref.	
Tertile 1	20 (5.1)	56 (23.7)	1.14	(0.66–2.22)	0.971	1.17	(0.60–2.29)	0.539
Tertile 2	26 (8.8)	59 (25.0)	1.16	(0.58–2.35)		1.26	(0.62–2.58)	
Tertile 3	21 (4.0)	58 (24.6)	1.16	(0.53–2.52)		1.22	(0.96–2.69)	

CI, confidence interval; COG, center of gravity; OR, odds ratio.

^*^Adjusted by country and maternal education.

Considering pathologically confirmed gliomas only (Table 5), no increases in ORs were observed in relation to cumulative call time or RF. Compared with the non-users, those in the highest tertile of cumulative call time and RF energy in the hemisphere, tumor, and COG of the tumor had adjusted ORs of 0.46 (95% CI, 0.10–2.21), 0.45 (95% CI, 0.09–2.29), and 0.28 (95% CI, 0.05–1.48), respectively.

**Table 5.** Conditional logistic regression analysis for the risk of glioma from among all participants

	Cases	Controls	Crude model			Adjusted model^*^		
	*n* = 46	*n* = 86	OR	95% CI	*P* for trend	OR	95% CI	*P* for trend
**Cumulative call times until one year before reference date**								
Non-users	12 (26.1)	22 (25.6)	Ref.	Ref.				
0.23–49.5 (hours)	14 (30.4)	19 (22.1)	1.25	(0.41–3.82)	0.592	1.18	(0.37–3.77)	0.200
56.1–238.1	10 (21.7)	24 (27.9)	0.58	(0.16–2.16)		0.64	(0.16–2.54)	
282.8–7,678	10 (21.7)	21 (24.4)	0.71	(0.65–3.02)		0.70	(0.16–3.03)	
**Hemisphere RF energy**								
Non-users	12 (26.1)	22 (25.6)	Ref.	Ref.				
Tertile 1	19 (22.1)	19 (22.1)	1.72	(0.55–5.40)	0.024	1.62	(0.50–5.26)	0.012
Tertile 2	24 (27.9)	24 (27.9)	0.28	(0.06–1.31)		0.30	(0.06–1.47)	
Tertile 3	21 (24.4)	21 (24.4)	0.46	(0.10–2.20)		0.46	(0.10–2.21)	
**Tumor RF energy**								
Non-users	12 (26.1)	22 (25.6)	Ref.	Ref.				
Tertile 1	15 (32.6)	210 (23.3)	1.63	(0.55–5.60)	0.090	1.38	(0.38–4.97)	0.149
Tertile 2	11 (23.9)	24 (27.9)	0.86	(0.24–3.05)		0.92	(0.25–3.40)	
Tertile 3	8 (17.4)	20 (23.3)	0.44	(0.09–2.07)		0.45	(0.09–2.29)	
**COG**								
Non-users	12 (26.1)	22 (25.6)	Ref.	Ref.				
Tertile 1	16 (34.8)	21 (24.2)	1.71	(0.48–6.03)	0.146	1.46	(0.40–5.39)	0.044
Tertile 2	11 (23.9)	21 (24.2)	0.85	(0.25–2.96)		0.88	(0.25–3.15)	
Tertile 3	7 (15.2)	22 (25.6)	0.28	(0.05–1.41)		0.28	(0.05–1.48)	

CI, confidence interval; COG, center of gravity; OR, odds ratio; RF, radiofrequency.

^*^Adjusted by country and maternal education.

## DISCUSSION

In this study of Korean and Japanese young people, we observed a slight elevated risk of all brain tumor among the heaviest users of mobile phones. The confidence interval was large, however, and there was little evidence of a dose-response relationship. ORs by level of RF energy were close to 1.0, again with no evidence of an association with dose. Analyses were performed by merging the Korean and Japanese data because the statistical power was insufficient unless the inclusion criteria were expanded to include cases with midbrain tumors, which were excluded from the MOBI-Kids international study, with relaxed residential matching criterion for controls.

The greatest strength of this study was our assessment of RF exposure from mobile phone use. The RF exposure algorithm was modified to consider the specific types of phones used in Korea and Japan, using modified phone-type classifications corresponding to the situations in Korea and Japan. In addition, exposure from extremely low frequency^26^ and cordless phone use (DECT) were not included in the risk analysis because we determined that it was negligible. We also included the APC values for the different Korean and Japanese mobile communication systems. Moreover, the algorithm used mobile phone categories that were more specific to the phones used in Korea and Japan, resulting in a more precise exposure estimation of the study population.

This study is limited in that, although 22 Korean and 36 Japanese brain tumor cases were included, sample size was sustantialy smaller than that included in the international study and the event rate remained low. The generally low exposure levels compared with those observed in the INTERPHONE study, along with the skewed distribution, limited the statistical power of our study. Recall bias for weighted mobile phone exposure were neglectable, since participants were not aware of the generation of their mobile phones. Extensive sensitivity analyses and sub-studies were conducted in the international MOBI-kids study, including evaluating possible bias related to recall^26^ and non-participants,^27^ as well as analyses focusing on specific pathological brain tumor types.^28^

The results of the present study are consistent with those of the CEFALO study by Aydin et al,^29^ in which mobile phone use was not associated with the risk of developing brain tumors among patients aged 7–19 years in four countries between 2004 and 2008 (OR 1.36, 95% CI, 0.92–2.02). In the INTERPHONE study, the cumulative mobile phone call times were divided into 10 groups; gliomas were more likely to occur in participants with versus without phone use (OR 1.40; 95% CI, 1.03–1.89).^4^ The same trend observed in the KJ study was demonstrated in that the remaining groups had lower risks of developing gliomas compared with participants without mobile phone use, although the number of participants with cumulative call times ≥1,640 hours in the group that used mobile phones was limited in the present study, which only investigated young people.

### Conclusions

Despite the aforementioned limitations, such as the small study sample size, our findings suggested that mobile phone use does not greatly impact the development of brain tumors. However, because we evaluated young people in both Korea and Japan who were geographically and genetically similar, small numbers and large confidence intervals might preclude any clear conclusions.

## SUPPLEMENTARY MATERIAL

eFigure 1.

eFigure 2.
