# Supplementary material for: Impact of Radiofrequency Exposure From Mobile Phones on the Risk of Developing Brain Tumors in Korean and Japanese Adolescents: A MOBI-Kids Case-control Study
Source: J Epidemiol. 2024 Apr 5;34(4):180–6. doi: 10.2188/jea.JE20230005 (PMC10918333; doi:10.2188/jea.JE20230005)
Supplement: Supplementary file 1 [file je-34-180-s001.pdf]

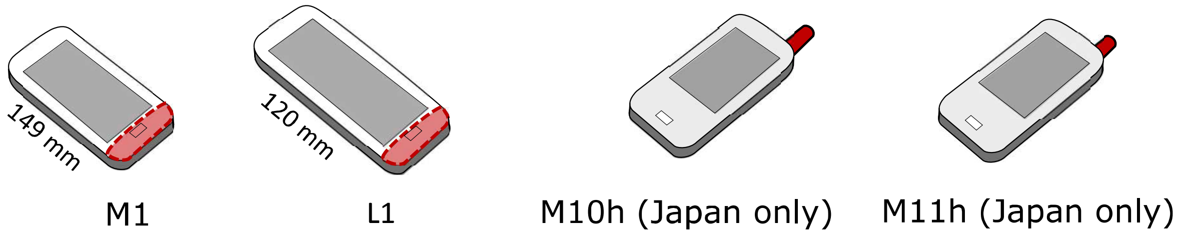

(**A**) Bar type

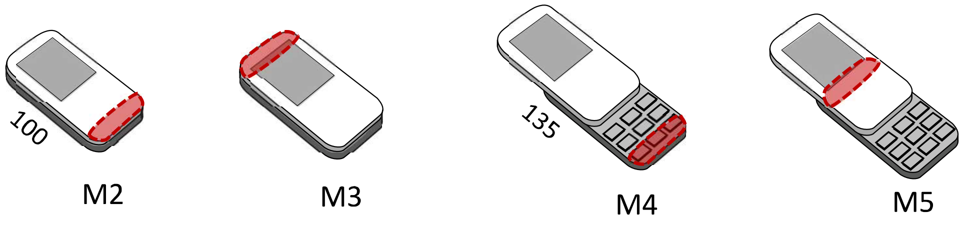

(**B**) Slide type

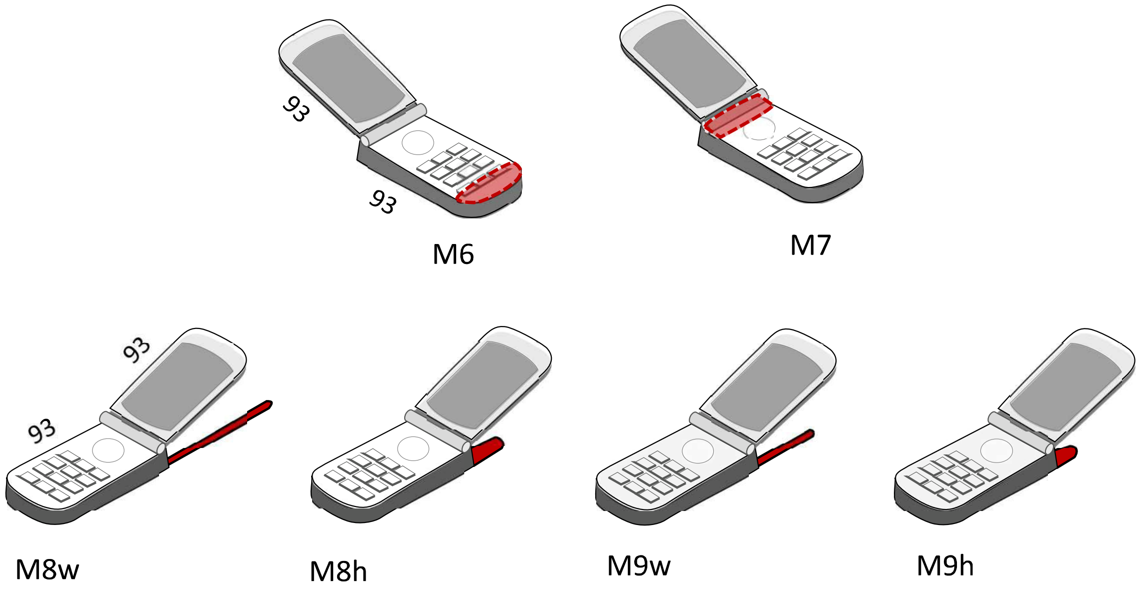

(**C**) Flip type

eFigure 1

$$CSE_l = \sum_{n=1}^{N} \left\{ \left[\begin{array}{l} \left(SAR_{l,\bar{t}_1,f,n,i}\Big|_{right} \cdot L_n + SAR_{l,\bar{t}_1,f,n,i}\Big|_{left} \cdot (1-L_n) \right) \cdot \dfrac{SAR_{\max 1}}{CF} \cdot c_1 \quad + \\ \left(SAR_{l,\bar{t}_2,f,n,i}\Big|_{right} \cdot L_n + SAR_{l,\bar{t}_2,f,n,i}\Big|_{left} \cdot (1-L_n) \right) \cdot \dfrac{SAR_{\max 2}}{CF} \cdot c_2 \end{array} \right] \cdot O_{f,s,o,u} \cdot \mathrm{DTx}_n \cdot T_n \cdot \mathrm{Days}_n \right\}$$

where, subscripts

l	cell location of reference brain
s	communication system (CDMA2000, WCDMA, ⋯)
n	the nth phone reported by a subject
t	commercial phone model
$\bar{t}$	numerical phone model
i	age
L_n	laterality factor (= right side-use factor)
T_n	call time during the nth phone use (sec/day)
$O_{f,s,o,u}$	APC modifier
$SAR_{\max 1}$	The maximum phone specific SAR of a commercial phone
CF	SAR conversion factor. 1.3 for 835 and 900 MHz and 1.6 for 1765, 1800, and 1850 MHz if Max SAR given for the averaging mass 1g.
c1 and c2	weighting factor applied to SAR data ($c2 \neq 0$ if slide phones or retractable antenna phones)
DTx	Discontinuous Transmission
Days_n	(Ageend-Agestart)*365.2

eFigure 2
